# Impact of acquisition volume on cone beam computed tomography imaging of marginal bone: an ex vivo study

**DOI:** 10.2340/aos.v83.40494

**Published:** 2024-04-25

**Authors:** Maurice Ruetters, Korallia Alexandrou, Holger Gehrig, Sinclair Awounvo, Ti-Sun Kim, Anna Felten, Christopher Lux, Sinan Sen

**Affiliations:** a Department of Conservative Dentistry, University Hospital Heidelberg, Heidelberg, Germany; b Department of Orthodontics, University Hospital Heidelberg, Heidelberg, Germany; c Institute of Medical Biometry, University Hospital Heidelberg, Heidelberg, Germany; d Department of Orthodontics, University Hospital Schleswig Holstein, Kiel, Germany

**Keywords:** Cone beam computed tomography, low-dose cone beam computed tomography, computed radiography, periodontitis, periodontal bone defects

## Abstract

**Objective:**

The current study explores whether there is a clinically relevant distinction in the measurement of marginal bone loss when comparing high-dose (HD) versus low-dose (LD) cone beam computed tomography (CBCT) protocols in small and large acquisition volumes.

**Material and Methods:**

CBCTs of four human cadaveric preparates were taken in HD and LD mode in two different fields of view 8 × 8 cm^2^ (LV) and 5 × 5 cm^2^ (SV). In total, 43 sites of 15 teeth were randomly chosen, and marginal bone loss was measured twice in all protocols at 43 sites of 15 teeth by one calibrated investigator. Bland-Altman plots and Lin’s concordance correlation coefficient (CCC) were calculated to assess the extent of agreement of the measurements. Additionally, the rater scored the certainty in each of the measurements.

**Results:**

For HD-CBCT CCC of measurements obtained using SV versus LV was 0.991. CCC of measurements obtained using SV versus LV of LD-CBCT was 0.963. Both CCC values indicated excellent agreement between the two volumes in both protocols.

CCC also indicated high intramodality correlation between HD-CBCT and LD-CBCT independent of the acquisition volume (0.963 – 0.992). Bland-Altman plots also indicated no substantial differences. Results of certainty scoring showed significant differences (*p* = 0.004 (LV), *p* < 0.001(SV)) between the LD and HD-CBCT.

**Conclusions:**

Accuracy of measurements of bone loss shows no clinical noticeable effects depending on the CBCT volume in this ex vivo study. There appears to be no relevant advantage of SV over LV, neither in HD-CBCT nor in LD-CBCT and additionally no relevant advantage of HD versus LD in visualizing marginal bone loss.

## Introduction

In recent years, the field of dental three-dimensional imaging has witnessed the introduction of an increasing array of cone beam computed tomography (CBCT) protocols, each designed to enhance diagnostic capabilities while addressing growing concerns about patient radiation exposure [1]. A significant thrust in this domain has been the development and optimization of strategies aimed at minimizing the radiation dose associated with CBCT. Among these strategies, the implementation of pre-programmed ‘low-dose’ (LD) protocols has emerged as a prominent approach [2]. The diagnostic efficacy of these LD protocols for a variety of clinical indications, such as the detailed visualization of peri-implant defects and complex periodontal structures, including furcations and marginal bone, has undergone extensive scrutiny and evaluation by multiple research groups. The outcomes of these investigations have been promising, suggesting that LD protocols could play a pivotal role in establishing a new standard for three-dimensional imaging, particularly in specialized fields like periodontology [3–6]. Furthermore, there are already initial studies that have investigated the suitability of low-dose digital volume tomography (LD-CBCT) in a clinical setting, for example, for the assessment of anatomical structures near wisdom teeth. Here too, the results were predominantly positive. The only exception was that the periodontal gap could be better determined in conventional CBCT protocols [7].

Concurrently, another significant approach to radiation reduction involves the strategic minimization of the acquisition volume [8]. This method not only achieves a decrease in radiation exposure but also leads to benefits such as reduced image noise and therefore enhanced image clarity [9, 10]. This aspect is particularly pertinent when imaging fine anatomical structures like the marginal bone, where precision is paramount. The visualization of the marginal bone holds substantial relevance in both periodontal and orthodontic treatment planning, as accurate knowledge of its morphology can aid in circumventing adverse effects like gingival recessions, which might arise from excessive tooth movements beyond the confines of the bony dental arch [11]. However, it is important not to choose volumes that are too small, in order to capture the regions of interest within a single image, thereby selecting a volume appropriate to the indication to avoid duplicate representations of the structures and the associated higher radiation exposure. Nonetheless, the question remains whether, in specific cases, a smaller volume might provide more detailed information regarding the marginal bone.

The aim of this study is therefore to validate whether there is a relevant advantage in illustrating marginal bone loss using small volumes (SV) compared to large volumes (LV) in LD as well as HD CBCT protocols.

Therefore, the hypotheses were:

1. Small-volume CBCT has no advantages over LV CBCT in depicting marginal bone loss, both in LD and HD protocols.
2. Low-dose CBCT is as suitable as HD CBCT for depicting marginal bone loss, both in small and large volume acquisitions.

## Materials and methods

This ex vivo study investigated 43 sites of 15 teeth from four human hemisected cadaveric heads. The number of different tooth types included in this study is listed in Table 1. Three of the teeth had amalgam restorations. The others were free of restorations.

**Table 1:** Tooth type and number.

Tooth type	number
Upper molars	5
Upper premolars	4
Lower molars	4
Lower premolars	2

**Figure 1:**
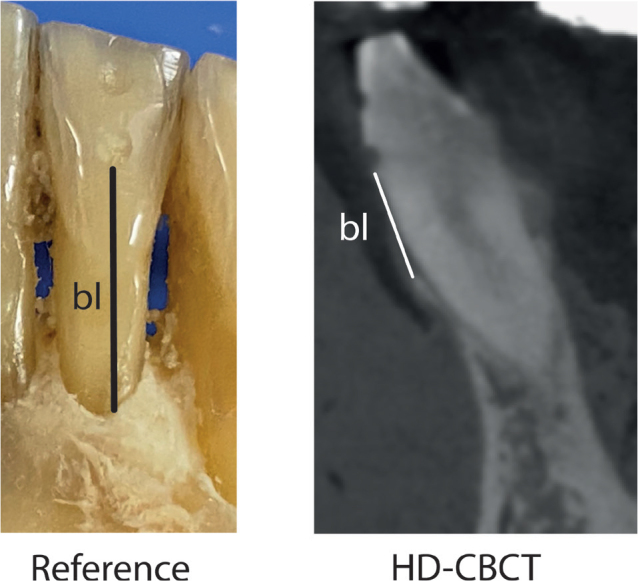
Measurement scheme. Left image (Reference): schematic image of the reference measurements bl. Right image (HD-CBCT): schematic image of the corresponding measurement in a HD-CBCT image. The orientation procedure was as follows: (1) the two depressions were identified, and the axis of the coronal plane was placed through the center of the depressions. (2) The axial slice was then aligned with the lower depression. (3) Measurements bl were then taken in the sagittal plane.

The heads were from bodies donated to the Institute of Anatomy and Cell Biology of the University of Heidelberg and were preserved with 99% ethanol, glycerin, and 37% formalin. At the time of the radiographic investigations, the hemisected cadaveric heads, including the mandibles, were fully covered by soft tissue and by the adjacent muscles of the cheek. The tongue, neck muscles, base of the skull, and cervical vertebrae were also still present. To ensure clear reproducibility of the image planes in the different acquisition modes, two depressions were made at the sites of the crown, where the measurements were performed by means of a round diamond burr (801L 314 016, Komet Dental, Gebr. Brasseler GmbH & Co. KG, Lemgo, Germany). The teeth were then radiographically imaged using two CBCT protocols (Figure 1): one LD-CBCT protocol and one high-dose (HD) CBCT protocol of one device (Veraview X800, J. Morita Europe, Dietzenbach, Germany) in the very same position.

The volumetric acquisition protocols were as follows:

- HD-CBCT protocol LV: 17.9 s radiation time, 5.00 mA, 102 kV, FOV 8 × 8 cm^2^, isotropic voxel size 0.125 mm, DAP 1396.95 mGy cm^2^.
- LD-CBCT protocol LV: 9.4 s radiation time, 1.6 mA, 72 kV, FOV 8 x 8 cm^2^, isotropic voxel size 0.125 mm, DAP 87, 19 mGy cm^2^
- HD-CBCT protocol SV: 17.9 s radiation time, 5.00 mA, 102 kV, FOV 4 × 4 cm^2^, isotropic voxel size 0.08 mm, DAP 383.57 mGy cm^2^.
- LD-CBCT protocol SV: 9.4 s radiation time, 1.6 mA, 72 kV, FOV 4 x 4 cm^2^, isotropic voxel size 0.125 mm, DAP 23.00 mGy cm^2^

The protocols were chosen because the field of view (FOV) of 8 × 8 allows for the depiction of the majority of the complete dentition. 4 × 4 was selected as it represents the smallest possible FOV of the available CBCT scanner. Additionally, the 8 × 8 protocol enabled comparability with previously conducted studies. The HD parameters were selected according to the manufacturer’s recommendations for an optimal image quality. For the LD protocols, the parameters were chosen to achieve the lowest possible radiation dose with the device. Analogous to previous studies, during imaging, gel pads were used to imitate the other half of the head to achieve tissue-equivalent volumes and ensure the most lifelike absorption of radiation [16]. The heads were fixed in position by placing the throat in a tube, and they were oriented in accordance with the orientation lines specified by the manufacturer.

### Probe measurements – reference standard

After radiological imaging, the gingiva was carefully removed by means of microsurgical instruments to ensure the bone was not damaged. Subsequently, in the axis of the previously milled depressions, the distance from the most apical point of the lower depression to the alveolar crest was measured on each site by means of a periodontal probe (Florida Probe, Clark Dental Equipment Systems Ltd, UK) with a 0.1 mm scale. Thus, a reference standard for bone loss (bl) measurements was established. These measurements were made by one experienced investigator (M. R.), who had previously been calibrated on a model. For calibration, the investigator had to successfully reproduce (relative agreement of 95%) the principal investigator’s (T. K.) bone-sounding measurements of clinical attachment loss at 168 sites on a standardized ex vivo reference model with a transparent gingiva (Co. M. Tech, Korea). These measurements are henceforth referred to as ‘probe measurements’ (Figure 1).

### Image review

For analysis, CBCT data was exported in DICOM format to the application software OSIRIX pro (aycanOsiriX 2.06.000). Windowing and levelling were allowed. Evaluations were all performed. All evaluations were performed on the same workstation and monitor (iMac, 27 in., Apple, California, USA) in the same dark room.

Measurement procedure has already been described in an earlier publication [5]. The orientation procedure was as follows: (1) The two depressions were identified, and the axis of the coronal plane was placed through the center of the depressions. (2) The axial slice was then aligned with the lower depression. (3) In the sagittal, measurements of bl were then taken in the sagittal plane. The images were reviewed by one calibrated dentist (K.A.) in multiplanar reconstruction. The measurement procedure is explained and shown in Figure 1.

For calibration of the method, the rater performed the measurements and segmentations in 20 teeth of different CBCT datasets to the ones of this study and discussed them with another highly experience rater (MR) with more than 10 years of experience in CBCT diagnostics till consensus was found.

Additionally, the rater scored the certainty in each of the measurements as ‘confident’, ‘diagnostically acceptable’, and ‘not confident’.

### Statistical analysis

For statistical analysis, the Lin’s CCC was calculated alongside 95% confidence intervals to assess the agreement between measurements obtained using LD-CBCT LV and SV as opposed to using HD-CBCT LV and SV as well as using LD-CBCT LV as opposed to using LD-CBCT SV and using HD-CBCT LV as opposed to using HD-CBCT SV. Bland-Altman plots were drawn separately per trial (method agreement) to graphically support the analysis of measurements agreement. Moreover, a Wilcoxon two-sample signed rank test was used to compare the measurements’ certainty of the rater between the modalities.

The analysis was performed using the statistical software R version 4.2.1.

## Results

**Figure 2:**
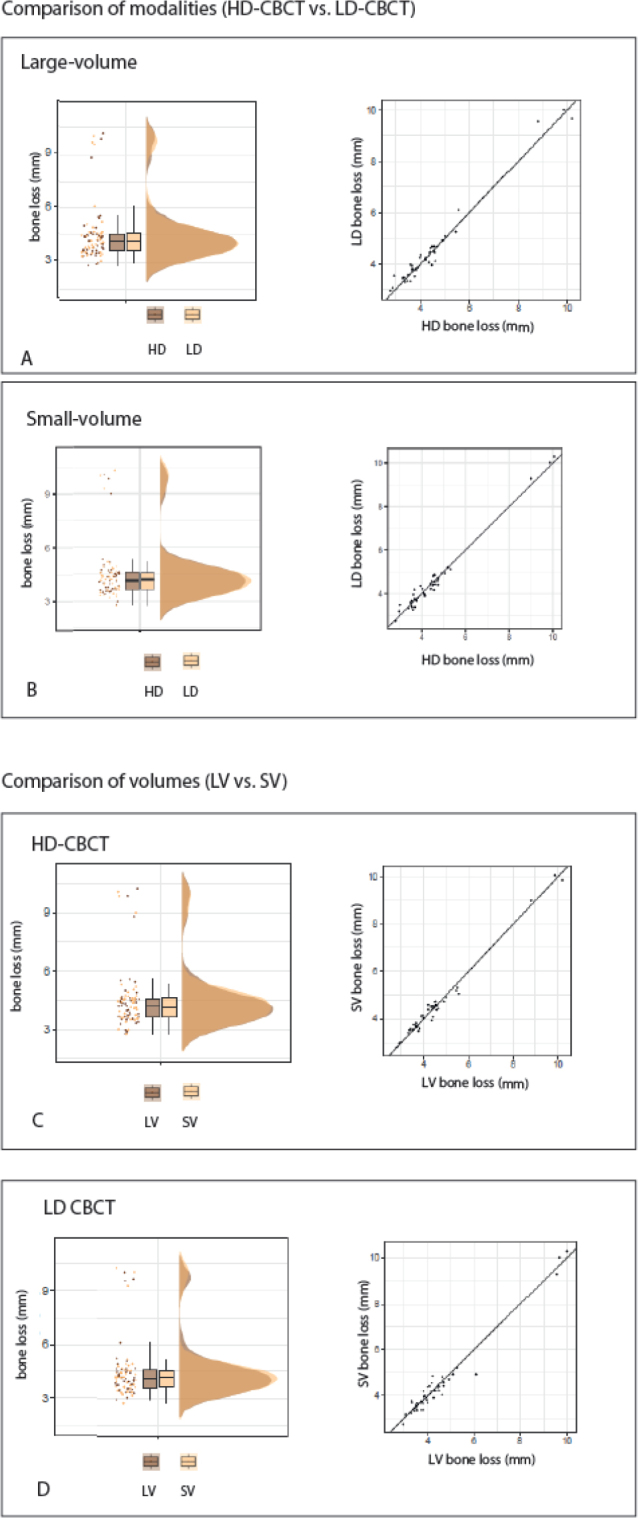
Descriptive statistics of the different modalities and volumes.

Figure 2A–D descriptively shows the distribution of marginal bone loss measurements of the different modalities and volumes.

Lin’s CCC of the different volumes compared with the reference measurements and Lin’s CCC of the comparison of different volumes are shown in Table 2.

**Table 2:** Lin’s concordance coefficients (CCC) of comparison of volumes with reference measurements and of different volume comparisons.

	CCC	Lower CI	Upper CI	interpretation
Ref vs. HD-CBCT LV	**0.992**	0.987	0.995	Almost perfect
Ref vs. LD-CBCT LV	**0.98**	0.967	0.988	Substantial
Ref vs. HD-CBCT SV	**0.993**	0.989	0.996	Almost perfect
Ref vs LD-CBCT SV	**0.991**	0.985	0.995	Almost perfect
HD-CBCT ***LV*** vs. LD-CBCT ***LV***	**0.969**	0.958	0.977	Substantial
HD-CBCT ***SV*** vs. LD-CBCT ***SV***	**0.992**	0.987	0.995	Almost perfect
HD-CBCT ***LV*** vs. HD-CBCT ***SV***	**0.991**	0.988	0.994	Almost perfect
LD-CBCT ***LV*** vs. LD-CBCT ***SV***	**0.963**	0.948	0.973	Substantial

CI = confidence interval, LV = Large volume, SV = Small volume.

**Figure 3:**
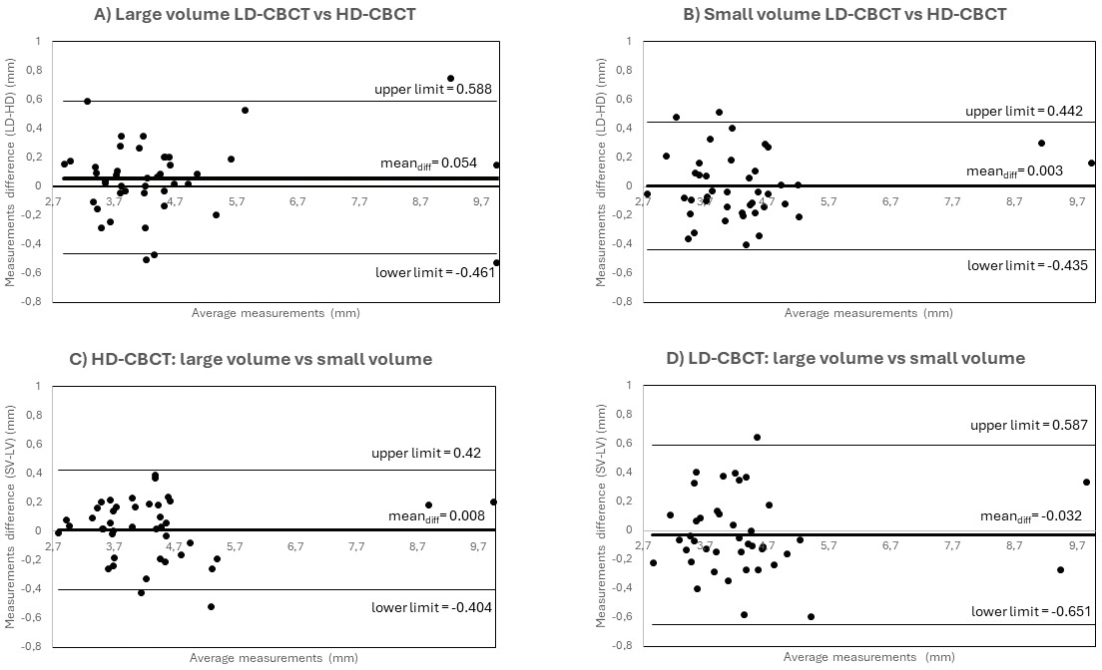
Bland-Altman Plots of (A) measurements of LV LD-CBCT versus HD-CBCT (B) measurements of SV LD-CBCT versus HD-CBCT (C) LV HD-CBCT measurements versus SV HD-CBCT measurements (D) LV LD-CBCT measurements versus SV LD-CBCT measurements.

Supporting Bland-Altman analysis is shown in Figure 3A–D. All means of measurements are around 0 and limits of 95% of agreements within clinically acceptable range (< 1 mm). Means of differences show no systematic over- or underestimation of any modality.

Results of certainty scoring are shown in Table 3. Significant differences were only shown between the LD and HD-CBCT but not depending on the CBCT volume.

**Table 3:** Comparison of means of certainty scoring of different measurements in different modalities.

	Modalities		*p*
HD-CBCT LV	vs.	HD-CBCT SV	
*1.628 ± 0.62*		*1.721 ± 0.50*	**0.384**
			
LD-CBCT LV	vs.	LD-CBCT SV	
*1.286 ± 0.67*		*1.023 ± 0.64*	**0.061**
			
HD-CBCT LV	vs.	LD-CBCT LV	
*1.628 ± 0.62*		*1.286 ± 0.67*	0.004*
			
HD-CBCT SV	vs.	LD-CBCT SV	
*1.721 ± 0.50*		*1.023 ± 0.64*	< 0.001*

* = significant.

## Discussion

The results of the study confirm both hypotheses that SV CBCT has no advantages over LV CBCT in depicting marginal bone loss, both in LD and HD protocols, and that LD CBCT is as suitable as HD CBCT for depicting marginal bone loss, both in small and large volume acquisitions. High Lin’s CCC values (> 0.9) show equal potential of LV compared to SV in both, HD-CBCT and LD-CBCT (Table 3). Bland-Altman Plots indicate means of measurements around 0 for measurements of marginal bone loss for both modalities and volume sizes showing that there is no substantial over- or underestimation of neither small nor large volume as well as LD-CBCT or HD-CBCT. The differences observed in measurements are all smaller 1 mm and within clinical tolerance values.

Lin’s CCC was also substantial to almost perfect for all modalities compared to the clinical references (Table 2). The results are in line with other studies that have addressed the suitability of CBCT for imaging dental structures. These studies have shown not only the potential of HD-CBCT but also the significant potential of LD-CBCT in depicting periodontal bone structures [2, 5, 6, 12].

Concerning the certainty of measurements, significant differences were only seen between LD-CBCT and HD-CBCT but not dependent on the volume (Table 4). Due to the subjectively perceived poorer image quality of LD-CBCT, these results are not surprising and in line with the results of an existing study by Charuakkra et al. [13]. However, the effects on the actual measurement results are not clinically relevant. The results confirm the assumption that more experience of the examiner is necessary for a better and confidenter interpretation of LD-CBCT images as already shown for endodontic tasks in HD-CBCT [14].

**Table 4:** Lin´s Concordance Coefficients (CCC) of comparison of volumes with reference 363 measurements and of different volume comparisons.

	CCC	Lower CI	Upper CI	interpretation
Ref vs HD-CBCT LV	**0.992**	0.987	0.995	almost perfect
*Ref vs LD-CBCT LV*	**0.98**	*0.967*	0.988	substantial
Ref vs. HD-CBCT SV	**0.993**	0.989	0.996	almost perfect
Ref vs LD-CBCT SV	**0.991**	0.985	0.995	almost perfect
*HD-CBCT* ***LV*** *vs. LD-CBCT* ***LV***	**0.969**	*0.958*	0.977	substantial
HD-CBCT ***SV*** vs LD-CBCT ***SV***	**0.992**	0.987	0.995	almost perfect
HD-CBCT ***LV*** vs HD-CBCT ***SV***	**0.991**	0.988	0.994	almost perfect
*LD-CBCT* ***LV*** *vs LD-CBCT* ***SV***	**0.963**	*0.948*	0.973	substantial

CI = confidence interval, LV = Large 364 volume, SV = Small volume.

### Limitations

The ex vivo nature of the experiments eliminated the risk of natural motion, such as tremors, which can lead to motion artifacts that can significantly reduce the quality and information content of the image [9, 15].

Only half heads were used. To mimic the missing half, gel pads were used as described in the Methods section. However, these pads cannot imitate natural bony structures, teeth, or restorative materials or the artifacts caused by these structures. This means that the image quality may have been slightly better than it would have been for complete heads [16].

## Conclusion

Accuracy of measurements of bone loss show no clinical noticeable effects depending on the CBCT volume in this ex vivo study. There appears to be no relevant advantage of SV over LV, neither in HD-CBCT nor in LD-CBCT and additionally no relevant advantage of HD versus LD in visualizing marginal bone loss. This means: choosing a smaller volume as well as HD protocols does not improve the assessability of marginal bone loss in either LD- or HD-CBCT. In accordance with European guidelines, it is clinically imperative to always employ the lowest feasible radiation dose to achieve optimal patient outcomes with respect to the indication [17]. In conclusion of the present findings, the smallest LD protocol possible in dependence of the indication should be utilized for the depiction of marginal bone loss whenever possible. For instance, to depict the entire dentition in periodontally diseased but still fully dentate patients, who require a CBCT scan before the initiation of therapy, an FOV 8 × 8 cm should be used. Conversely, for planning surgically complex procedures on a single tooth involved in furcation, a small field in low dose would suffice. However, it should be noted that this is an in vitro study, and the results need to be confirmed in vivo, not least because of the limitations discussed previously.
